# Self-Propelling Water Droplets on Conical Spikes with Sawtooth Surface Structure

**DOI:** 10.1021/acsami.5c03846

**Published:** 2025-05-16

**Authors:** Abubaker S. Omer, Aikifa Raza, TieJun Zhang

**Affiliations:** Department of Mechanical and Nuclear Engineering, 105955 Khalifa University of Science and Technology, P.O. Box 127788, Abu Dhabi, United Arab Emirates

**Keywords:** capillarity, directional transport, fog harvesting, sawtooth structure, self-propulsion, surface wettability, 3D printing

## Abstract

Directional fluid transport is critical for water, energy, and biomedical applications, including passive fog harvesting. The unique shape gradient of conical structures can induce capillary pressure and drive the self-propulsion of droplets as the droplets settle on wettable sharp cones and move toward the cone base as they grow. In this work, we achieve passive droplet transport by fabricating conical spikes with sawtooth and imbricated (reversed-sawtooth) surface structures via high-resolution 3D printing. Fog harvesting experiments on various spikes indicate that the sawtooth structure exhibits the most efficient droplet mobilization toward the spike base, while the imbricated surface structure promotes isolated droplet formation with delayed transport and the smooth spikes would keep droplets stationary unless coalescences occur. Further droplet motion analysis reveals that the flat surface with imbricated structure exerts 3.5 times more hysteresis force than the sawtooth one under dry conditions and nearly twice under wet conditions. During fog harvesting, microdroplets in fog fill the teeth gaps along the water-wet sawtooth spike, and the resulting big barrel droplet exhibits a series of stop-and-go motions when it continues growing. Our quantitative analysis reveals that the interplay between the capillary and hysteresis forces is responsible for the droplet self-propulsion. Our experiments with the conical sawtooth spike array further demonstrate that the fog water harvesting rate with 10 μm teeth spacing is twice that with 20 μm spacing and triple that with 40 μm spacing.

## Graphical abstract

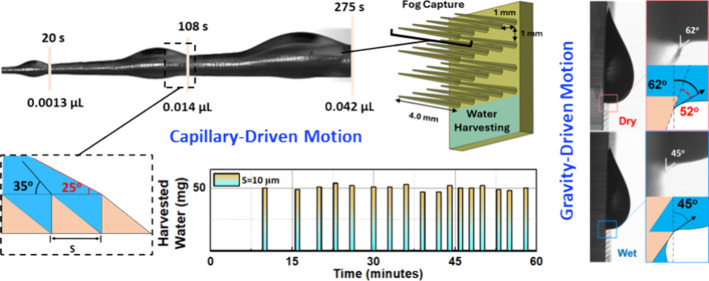

## Introduction

Directional fluid transport plays a crucial role in many water, energy, and biomedical applications, such as fog harvesting,^1–3^ self-cleaning surfaces,^4,5^ and enhanced heat transfer.^6–8^ These surfaces are often designed and engineered to mimic the nature as these bioinspired surface structures can leverage micro- and nanoscale features to induce favored fluid movement and passive transport.^9–11^

There are three types of forces for passive droplet transport along a certain direction: body forces, energy gradient, and curvature gradient. A common body force-driven example is that droplets slide down the inclined or vertical surfaces due to weight, where gravity overcomes the lateral interfacial adhesion force.^12,13^ Additionally, the energy gradient, also known as the wettability gradient, relies on altering the surface either topographically,^14–16^ chemically,^17,18^ or both^19,20^ to direct the fluid motion. This manipulation causes the fluid to move toward areas with higher surface energy. Furthermore, the force generated from the curvature gradient arises from the interaction between the droplets and surfaces with varying cross sections, such as pore confinement,^21^ diverging channel,^22^ and conical shape.^23,24^ This interaction changes the curvature across the droplet profile, making it move along the direction of minimizing surface area. This mechanism of directional fluid transport is ubiquitous in nature^25–27^ (Figure 1a) and inspires many applications, particularly in fog collection.

**Figure 1:**
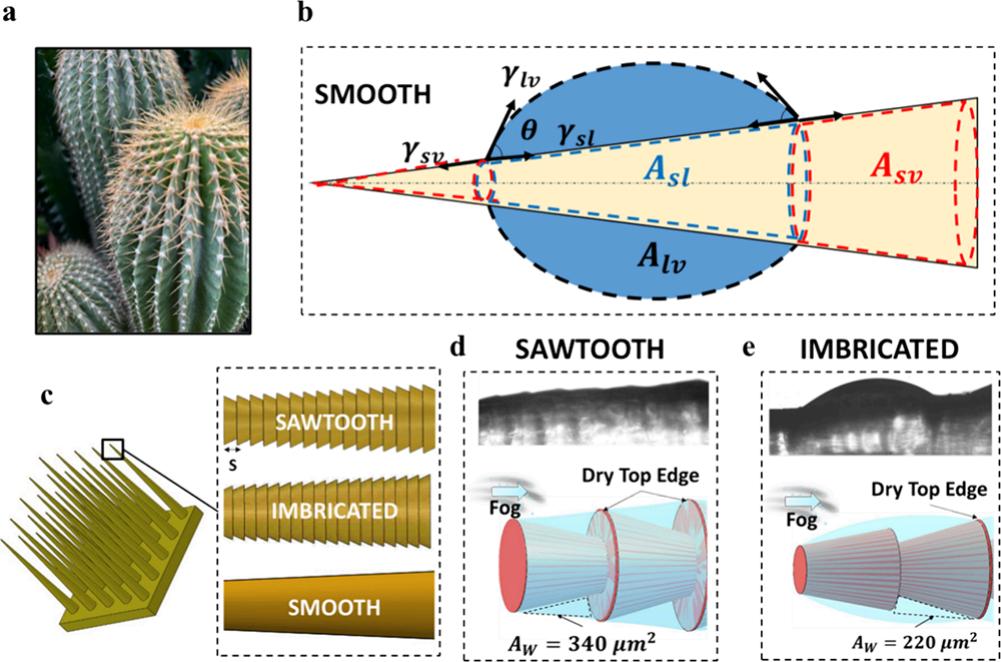
Surface-Structured Spikes for bioinspired fog harvesting. (a) Optical image of the Cactus plant with conical spikes that enable fog harvesting. (b) Schematic of a microdroplet wetting a conical surface. (c) Schematics showing the bioinspired design of a 3D-printed array of spikes and the tested spike types (sawtooth, imbricated and smooth). (d) Wetting of the sawtooth surface structure during fog exposure. (e) Wetting of the imbricated surface structure during fog exposure.

The utilization of conical surfaces to achieve capillary-driven droplet transport is a well-established principle in fog harvesting research. Studies employing biomimetic approaches have significantly contributed to this field. For instance, conical microknots of spider silk, in conjunction with a wettability gradient, enable passive droplet transport through capillary mechanisms.^25^ Moreover, hierarchical microgrooves on cactus spines facilitate fog harvesting through capillary-driven transport.^26^ These biological studies have inspired the design and fabrication of numerous fog harvesting devices. Building upon these principles, conical surfaces with hydrophobic,^23^ superhydrophilic^28,29^ or gradient^30^ surface wettability have been fabricated and demonstrated significant potential in enhancing fog harvesting performance.

Micro/nanostructured surfaces, often combined with chemical coatings, are also utilized to control droplet motion and enhance the efficiency of atmospheric water harvesting. Ghosh et al. improved dropwise condensation heat transfer by enabling capillary-driven condensate removal through the creation of superhydrophilic conical regions on a hydrophilic substrate.^31^ Qi et al. developed hydrophilic patterned slippery liquid-infused porous surfaces (SLIPS) on copper substrates to facilitate efficient droplet transport during fog harvesting.^32^ Fu et al. fabricated conical microneedles combined with strips exhibiting contrasting wettability (superhydrophilic and superhydrophobic), integrating them into one-dimensional spiral copper wires.^33^ These studies demonstrate the key role of engineered surface topographies and wettability patterns in optimizing droplet transport for enhanced water harvesting practices.

Furthermore, the use of open channels offers distinct advantages in enhancing droplet transport.^34^ Inspired by scallop shells, Haoyu et al. engineered a surface featuring superhydrophilic V-shaped channels to improve fog harvesting and enable unidirectional transport, achieving a maximum flux of 450 mL·h^–1^.^35^ Drawing inspiration from a vampire bat’s tongue, Ye et al. designed a superhydrophilic flexible origami surface with parallel-arranged shaped channels, reaching a peak liquid transport speed of 65 mm·s^–1^ and a liquid retention capacity 4.6 times greater than that of a conventional superhydrophilic sheet.^36^ Zhang et al. developed a surface featuring serial wedge-shaped superhydrophilic patterns capable of promoting instantaneous droplet velocity of 207.7 mm·s^–1^.^37^

In this work, we propose to employ conical spikes with periodic surface structures shown in Figure 1c, for enhancing the mobilization of growing water droplets, which are captured by the sharp spikes from a fog stream. As these droplets grow along spikes, their elevated energy state, coupled with the unique sawtooth structure on spike surface (Figure 1c), facilitates efficient self-propulsion of water droplets and enables high-performance fog harvesting with a dense array of conical spikes.

## Results and Discussion

Considering a droplet wetting a conical spike surface, the Gibbs surface free energy of this system is given by eq 1. For droplets smaller than the capillary length, the gravity effect, represented by the last term of eq 1, becomes negligible with microdroplet forming on spike as in Figure 1b. The change in the net surface free energy is given by *dE* = (γ_*lv*_ *cos* θ + γ_*sl*_ – γ_*sv*_) *dA*_*sl*_, where *dA*_*sl*_ is the change in the solid–liquid interface area and θ is the intrinsic water contact angle at the cone surface. Under equilibrium conditions, the system’s energy *E* is minimized with respect to the solid–liquid interface area as in eq 2, which is known as the Young-Dupree equation. In real scenarios, when a spherical microdroplet settles at the apex of a conical spike surface, it undergoes a shape transition that increases its liquid–vapor interfacial area, thereby elevating its energetic state. Equation 3 represents how the excess surface free energy is converted into work received by the droplet-cone system.^38–40^ This work causes the droplet to self-propel toward regions with larger conical radii, and it eventually stops at a location determined by the contact angle θ, droplet volume, and the cone’s apex angle. It is worth noting that larger droplets tend to migrate further toward the cone’s base.^41^

$$E=\gamma_{lv}A_{lv}+\gamma_{sl}A_{sl}+\gamma_{sv}A_{sv}+\rho gzV$$ 1

$$\gamma_{lv}\cos\theta +\gamma_{sl}-\gamma_{sv}=0$$ 2

$$F_{c}=-dE/ds$$ 3

where *E* represents the Gibbs surface free energy, γ interfacial tension, and *A* interface area. *l*, *v*, and *s* denote liquid, vapor, and solid phases, respectively. *F*_*c*_ is the capillary force, resulting from surface energy minimization, to drive droplet motion, and *ds* is the displacement along the cone’s generatrix.

Shape-gradient conical spikes (2α ≈ 4 °) with microscale sawtooth and imbricated structures, as shown in Figure 1c are fabricated by using high-resolution projection stereolithography 3D printing. High-magnification optical imaging was applied to investigate the growth and dynamics of droplets on the spikes under fog exposure. Though both the sawtooth and imbricated surface structures (i.e., microcavities, sharp edges) facilitate water nucleation and condensation along the spikes^42^ (Figure S1), they differ significantly in their orientation relative to the fog stream. The sawtooth structure presents vertical sharp edges that directly oppose the oncoming fog (Figure 1d), while the imbricated structure has inclined edges parallel to the flow (Figure 1e). This key difference in orientation, along with variations in the teeth interspacing area, contributes to the distinct motions of droplets on each surface structure when intercepting the fog.

A careful observation has indicated a distinct wetting behavior of microscale sawtooth structures throughout all the fog harvesting experiments. Initially, fog droplets fill the gaps among the teeth of wettable spikes, while the spreading water film is ruptured by the sharp edges of the sawtooth surface structure (Figure 1d). Subsequently, a barrel-shaped droplet forms near the spike tip, growing through coalescence with fog droplets, as depicted in time-lapse optical images in Figure S2. When the droplet’s front contact line expands to reach an apparent contact angle (θ_*app*_) of approximately 35 °, it self-propels toward the areas with larger local spike radii (*r*) in the stop-and-go motion, as shown in Figure 2b (Movie S1). After each self-propulsion event, the droplets’ coordinates (*x*_*1,*_ *x*_*2*_ and *x*_*3*_ seen in Figure S3a) are directly proportional to the radius (*R*) of a sphere with the same volume as the barrel droplet (Figure S3). Typically, a single barrel droplet forms and travels along the cone as it grows (Figure 2a). In fact, multiple droplets can form and coalesce, either moving toward the larger conical radius (Figure 2c) or draining into larger stagnant droplets (Figure 2d). Ultimately, all events ended in a single large barrel droplet progressing slowly toward the broader base of the spike. Sawtooth spikes with varying teeth spacing (10, 20, and 40 μm) were fabricated (Figure S4), and all exhibited similar droplet dynamics. Notably, droplets on the spike with 10 μm teeth spacing (S-10) covered a larger solid–liquid area compared to those with 20 and 40 μm teeth spacing (S-20 and S-40), demonstrating the potential of enhancing droplet mobility and fog harvesting efficiency with decreased sawtooth spacing (Figure S3c).

**Figure 2:**
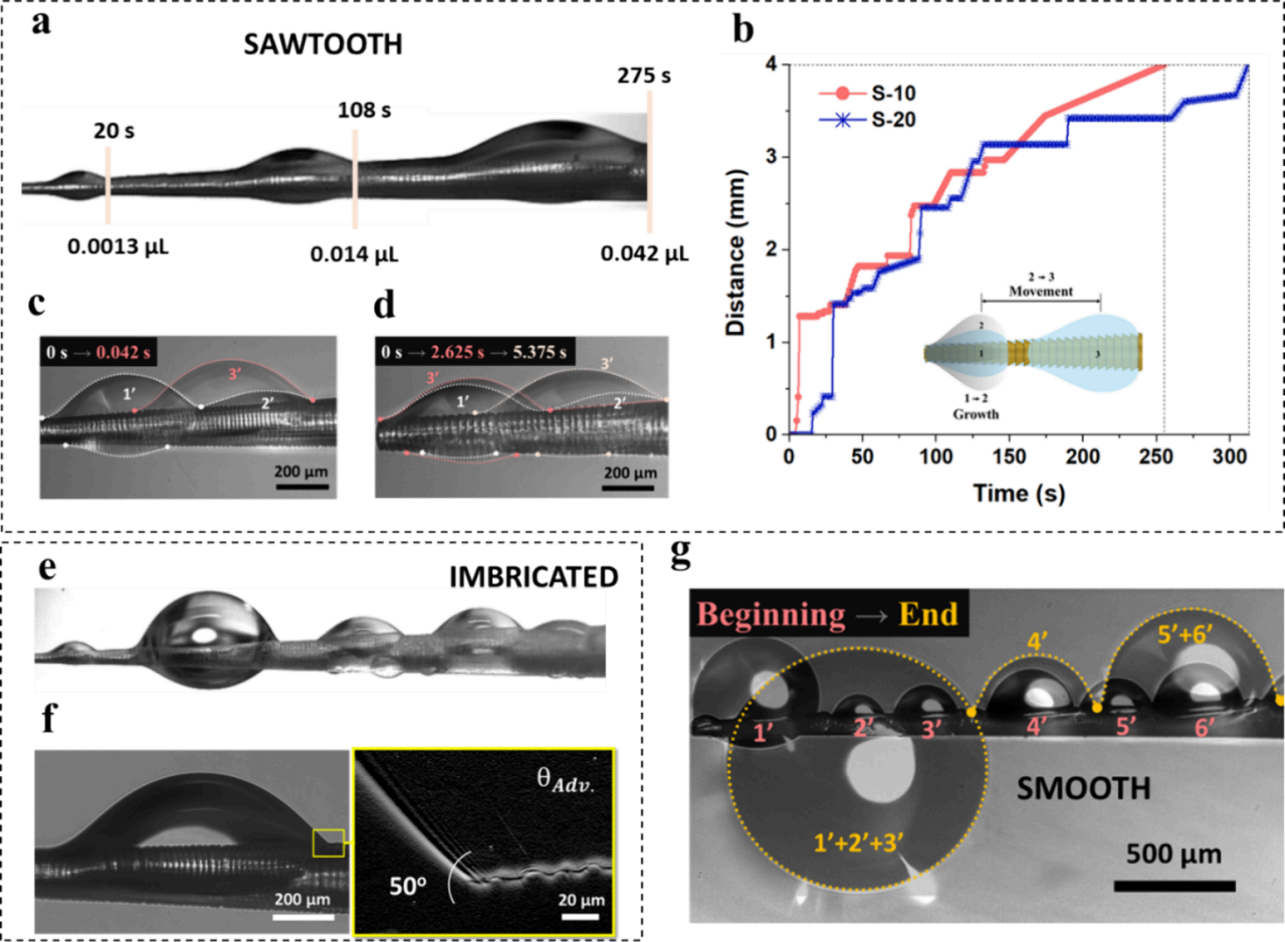
Overview of fog harvesting and the associated droplet dynamics. (a) Time-lapse images of a droplet growing and progressing on the S-20 sawtooth spike. (b) The typical journey of a single barrel droplet progressing along the S-10 and S-20 sawtooth spikes. (c) Coalescence-induced self-propulsion. (d) Drainage of a smaller droplet into a larger one, followed by self-propulsion resulting from coalescence-induced growth. (e) Isolated large droplets forming on the imbricated spike. (f) Advancing contact angle of the droplet on the imbricated spike. (g) Droplet coalescence and immobilization on the smooth spike.

Imbricated spikes with reversed sawtooth orientation were fabricated (Figure S5) and exposed to fog for similar experiments as sawtooth for comparison. The spikes captured microdroplets from fog that filled the cavities initially, while isolated large droplets formed at the surface structure ultimately, as shown in Figure 2e. These droplets eventually exhibited two kinds of motions: coalescence-induced and stop-and-go movements (Movie S2). In areas without neighboring droplets, the advancing angle was approximately 50° and the receding angle was near zero, as shown in optical image in Figure 2f. Eventually, it took longer time for droplets on the imbricated surface structure to reach the conical spike base than those on sawtooth one (Figure S6).

To further evaluate the role and efficacy of the spike surface structures, directional droplet transport experiments were also performed with smooth glass spikes (2α ≈ 3°). The glass spike exhibited a water contact angle of 30°, and remarkable coalescence-induced self-propulsion during fog harvesting (Figure S7). To ensure a fair comparison, the commercial glass spikes were coated with 3D printing resin to maintain the same surface wettability as the 3D-printed structured spikes (Figure S8). The result in Figure S8c shows that multiple hemispherical droplets with a contact angle of 49° form along the smooth spike’s axis during fog harvesting experiment. These droplets coalesce but do not mobilize, remaining largely stagnant throughout the experiment (Figure 2g) (Movie S3). Slight contact line movements revealed an advancing angle of 77° and a receding angle of 10° (Figure S8d – e). Overall, our results demonstrate enhanced fog harvesting with sawtooth spikes as they display smaller, more mobile droplets, indicating superior performance compared to the spikes with smooth surfaces and imbricated structures.

The barrel-shaped droplets on the sawtooth spikes exhibit a consistently hyperenergetic state as they grow through coalescence with fog droplets, indicating a significant influence from the characteristics of the fog stream. Our experimental setup utilized an ultrasonic atomizer with a mass flow rate of 0.34 mg/s (Figure S9) to generate fine fog droplets (Figure 3a). Based on the liquid atomization principle, high-frequency ultrasound induces unstable capillary waves and generates mist with droplet sizes determined by the wavelength of these waves.^43–45^ We adopted the droplet size distribution (Figure S9d) for a 2.4 MHz frequency atomizer,^43^ which is similar to our experimental setup. This allowed us to calculate the droplet size distribution per cubic millimeter of air (Figure 3b), as well as the total surface area and volume of the fog droplet population. By studying the surface energy increase of the barrel droplets during stagnation periods prior to self-propulsion events, we ensured that no energy minimization occurred during the calculation periods. Our results have demonstrated that the rate of surface energy increase in the barrel droplet is generally consistent with the combined energy of the coalescing fog droplets (Figure S9e).

**Figure 3:**
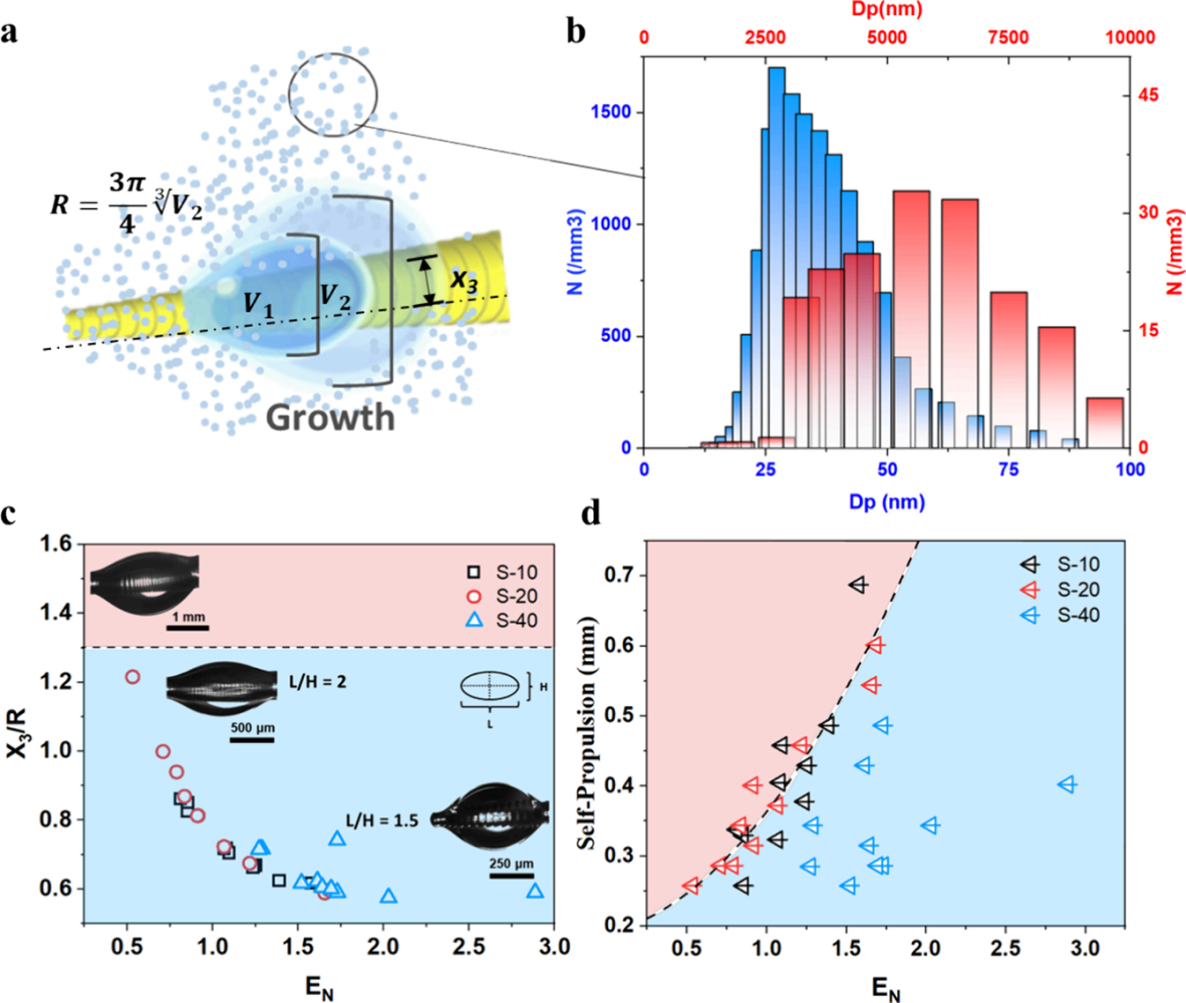
Multiscale energy analysis from fog stream to droplet self-propulsion. (a) Schematic diagram of barrel droplet growth leading to self-propulsion on the sawtooth spike during fog harvesting: initial droplet volume (*V*_1_), droplet volume at self-propulsion (*V*_2_), equivalent radius (*R*), and coordinate (*x*_3_). (b) Fog droplet size distribution in number/mm*^3^* for a 2.4 MHz frequency atomizer. (c) Actual-to-spherical surface energy ratio before self-propulsion (*E*_*N*_) as a function of the normalized coordinate (*x*_*3*_ */R*), with the blue region representing the range where self-propulsion movements are possible, and the red region representing droplet stagnation due to gravity force overcoming the droplet’s excess surface energy, preventing self-propulsion. Optical images depicting the droplet’s configuration as it flattens and eventually rolls down due to gravity. (d) Distance of self-propulsion as a function of *E*_*N*_ with arrows pointing to the direction of energy minimization during self-propulsion, and the red region corresponds to stagnation where the droplet lacks sufficient surface energy for self-propulsion, while in the blue region, the self-propulsion is possible.

When the barrel droplet encounters a poorly wetted region, it requires extra surface energy (*E* = γ­(*A*_*lv*_ – *A*_*sl*_ *cos*θ) to move further. To investigate the relationship among droplet energy, size, and location, the surface energy of the barrel droplets was normalized to that of an equivalent spherical droplet with the same volume (*E*_*N*_ = *E*/γ4π*R* ^2^). The energy ratio *E*_*N*_ is correlated to the ratio between the droplet coordinate (*x*_*3*_) and the radius (*R*) of the equivalent spherical droplet. Figure 3c reveals three main characteristics: First, a universal curve encompasses the three sawtooth spikes, indicating that the energy ratio (*E*_*N*_) primarily depends on droplet size and location rather than the teeth spacing. Second, *E*_*N*_ is highest at the start of the fog harvesting experiments when the droplet occupies a small solid–liquid area, and the teeth gaps are not saturated with water. This is particularly prominent for S-40, as its larger gaps are harder to fill with water, resulting in a higher *E*_*N*_ compared to S-20 and S-10. Third, as the droplets grow and occupy larger conical areas, their hyperenergetic state decreases, leading to elongation and flattening of the droplet shape. Several studies have reported the flattening of barrel droplets at larger conical radii.^46,47^ This phenomenon is evident from the higher scalability of *x*_*3*_ compared to *x*_*1*_ as *R* increases (Figure S3c). As the droplet grows beyond the capillary length, gravity becomes the dominant force, driving the big droplet to roll downward and ultimately cease its movement. Additionally, Figure 3d demonstrates a positive correlation between the distance traveled during self-propulsion and *E*_*N*_. Results show that the self-propulsion of S-20 and S-10 approaches a joint trend, while for S-40 self-propulsion behaves more randomly and falls short due to insufficient wetting of teeth gaps. Furthermore, self-propulsion speed diminishes in larger, more wetted areas of the sawtooth spike. In these regions, the flatter droplets exhibit progressively slower motion, eventually coming to a complete stop and rolling due to gravitational forces overcoming capillary forces.

Capillary pressure and force analysis of barrel droplets revealed the physical insight of enhanced droplet mobility on sawtooth structured spikes. At any given point on the profile of a droplet wetting the sawtooth spike, there are two principal curvatures: one lies in the plane of the figure and the other in the perpendicular plane. As depicted in Figure 4a, the principal radii of droplet curvatures toward the tip (*R*_1_^*t*^ and *R*_2_^*t*^) are smaller than those toward the base (*R*_1_^*b*^ and *R*_2_^*b*^). This unevenness in curvature generates a capillary pressure gradient, with a higher pressure difference (Δ*P* ^*t*^) at the rear than the front (Δ*P* ^*b*^). Consequently, the droplet experiences a net force driving its axial movement toward the base region with a larger conical radius. Michielsen et al.^41^ proposed a model for a symmetrical barrel droplet on a cone, dividing it into two constant-pressure segments (a-b and b-c), as shown in Figure 4a. Capillary pressures at these segments can be calculated based on dimensionless numbers *n* ^*t*^ = *x*_2_/*x*_1_ and *n* ^*b*^ = *x*_2_/*x*_3_, cone apex angle (α) and water contact angle (θ_*app*._ *).* Alternatively, the sum of forces applied on the droplet at its apex must be equal to or less than zero for a stationary droplet. Forces for the (a-b) segment include: (i) the capillary force integrated over the droplet contour (*f*_1_ = 2π*x*_2_γ), (ii) the capillary force of the pressure (*f*_2_ = – Δ*P* ^*t*^π­(*x*_2_^2^ – *x*_1_^2^)), and (iii) the force exerted by the conical surface (*f*_3_ = – 2π*x*_1_γ), as shown in Figure 4b.^48^ Similarly, for the (b-c) segment, *x*_1_ is substituted with *x*_3_. Through image processing, the principal radii of the curvatures were measured from live footage. These measurements were then compared to pressure calculations using two distinct models: M1, based on Michielsen et al.’s work,^41^ and M2, based on the force balance at the droplet apex.^48^ Our results demonstrated good agreement between the measured and predicted curvature radii, validating these models’ applicability for describing droplet behavior on sawtooth conical surfaces (Figure S10).

**Figure 4:**
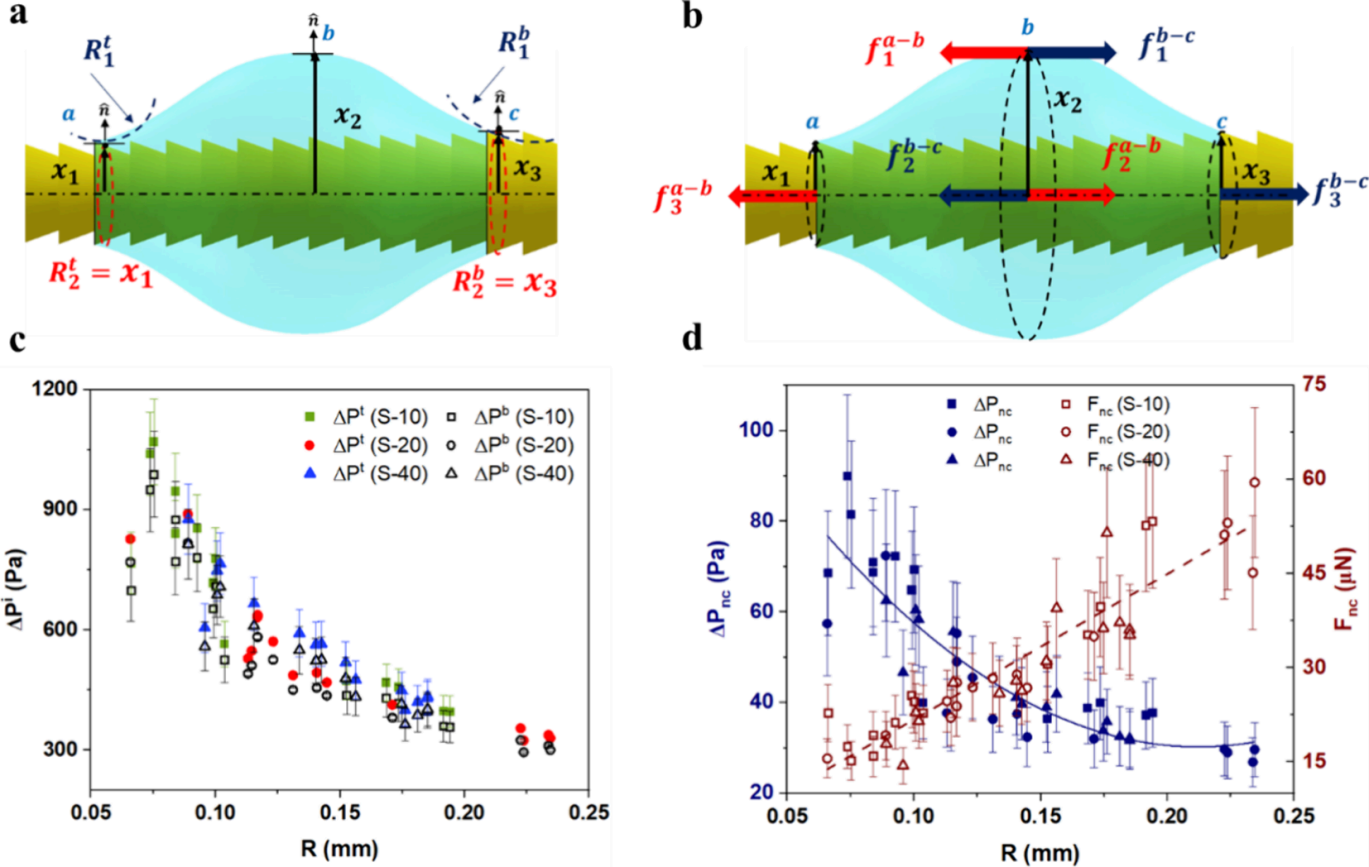
Capillary pressure and force analysis of barrel droplets on sawtooth structured spikes. (a) Schematic representations of the principal radii of curvatures and (b) the force balance at the droplet apex, with the red arrows and blue arrows depicting the forces on the (a-b) and (b-c) segments, respectively. (c) Capillary pressures at the rear (Δ*P* ^*t*^) and front (Δ*P* ^*b*^) ends of the droplet as a function of its spherical radius (*R*). (d) Net capillary pressure difference (Δ*P*_*nc*_) and force (*F*_*nc*_) as a function of *R*.

As the droplet grows, its principal radii of curvature increase, leading to a decrease in capillary pressure at both ends of the droplet due to the inverse relationship between pressure and radius. A comparison in Figure 4c of stagnant droplets on the three sawtooth spikes (S-10, S-20, and S-40) revealed that capillary pressure is primarily influenced by the droplet size, not saw-teeth spacing. Additionally, the net capillary pressure difference (Δ*P*_*nc*_) between the rear and front sides of the droplet was observed to decrease as the droplet grew. For larger and flatter droplets, Δ*P*_*nc*_ is significantly reduced, but as long as the droplet remains adhered to the conical surface, Δ*P*_*nc*_ persists due to the varying solid-surface curvature along the spike’s profile. Figure 4d demonstrates that as the droplet size increases, the surface area also grows, leading to an increase in net capillary force *F*_*nc*_ in eq 6.

$$\Delta P^{i}=\gamma_{LV}\left[\frac{1}{R_{2}^{i}}-\frac{1}{R_{1}^{i}}\right]$$ 4

$$\Delta P_{nc}=\Delta P^{t}-\Delta P^{b}$$ 5

$$F_{nc}=\Delta {P_{nc}}^{*}A_{lv}$$ 6

where *i* denotes *t* or *b*, indicating the rear and front sides of the contact line, respectively. Note that eq 4 can be calculated by applying the validated models M1 or M2 (eqs S5–S6).

As discussed previously, the increase in the barrel-shaped droplet’s surface area (*A*_*lv*_) leads to an increase in the capillary force (*F*_*nc*_), eventually overcoming the local hysteresis force (*F*_*h*_). This initiates self-propulsion, driven primarily by capillary forces and moderated by the counteracting hysteresis forces. The latter *F*_*h*_ arises from the meniscus interaction with the wetted gaps and dry tips of the sawtooth microstructures. This force balance generates a net acceleration force (m*dv/*dt*) in the nano-Newton range due to the droplet’s small mass, significantly lower than the micro-Newton range of the underlying capillary and hysteresis forces. When the droplet grows, the contact line’s front edge pins to the dry tip of a microtooth. As the droplet expands, this pinned contact line stretches until the tension overcomes the pinning force, causing it to snap at an angle θ_*Front*_ and snap forward toward the next sawtooth. The contact line then reaches a new angle θ_*Adv*._ due to the presence of water trapped ahead in the gap between teeth (Figure 5a). This process of pinning, stretching, and snapping repeats as the contact line encounters subsequent microteeth, generating a tension force per unit length (τ_*Front*_) directed toward the spike base. Conversely, for the droplet to move forward after the front-line snaps, the rear-line edge becomes pinned. It stretches and its angle reduces from θ_*Rear*_ to the receding contact angle θ_*Rec*._, generating a pinning tension force per unit length (τ_*Rear*_) in the opposite direction (Figure 5b). These opposing tension forces at both ends of the contact line create a net hysteresis force per tooth (*F*_*h*_), as represented by eqs 7–8. This force acts normal to the contact width between the droplet and the sawtooth spike. For a symmetrical barrel droplet, this contact width corresponds to the local spike perimeter (2π*r*) at the droplet’s apex.^49^ The magnitude of the capillary force is comparable to the hysteresis force, which is proportional to the number of teeth the droplet encounters during its self-propelled movement.^50^ Moreover, continuous droplet movement on sawtooth spikes is critically dependent on the wetted teeth gaps (Figure S11). Consequently, when a 2° apex angle spike exhibited poor wetting, barrel droplet transport was significantly hindered, demonstrating the vital role of effective wetting for efficient transport (Figure S12).

**Figure 5:**
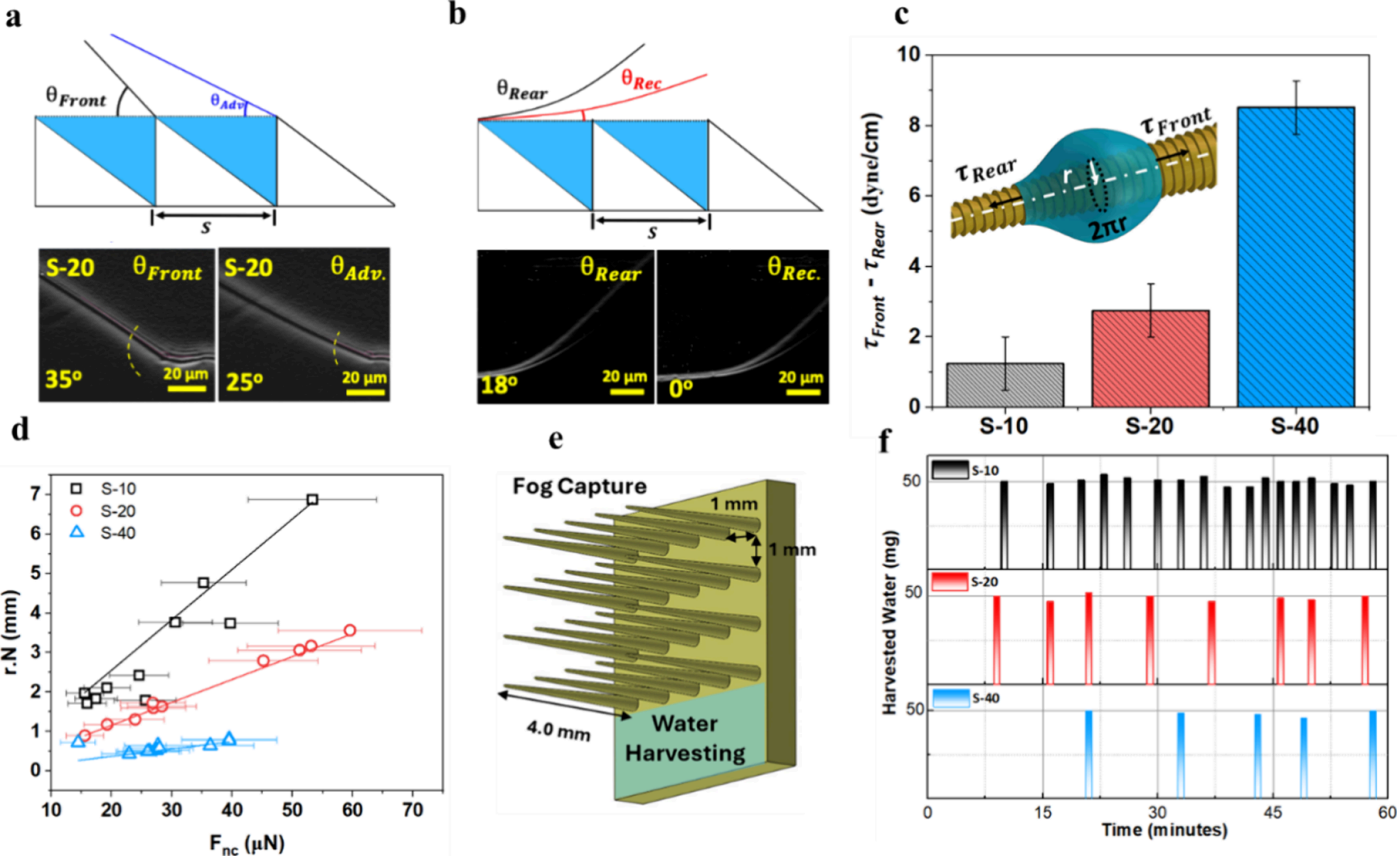
Fog harvesting with sawtooth spikes. Localized meniscus analysis of a droplet moving on a sawtooth spike, showing (a) contact line snapping at the front and (b) tensioning at the rear, propelling the droplet toward the base. (c) Net hysteresis force per unit width per tooth for barrel droplets on sawtooth spikes. (d) Predicted (lines) and measured values of *r*·*N* as a function of capillary force at the moment of self-propulsion (*F*_*nc*_). (e) Schematic of the 3D-printed array of spikes with an extended surface for water harvesting. (f) Fog harvesting rates of the sawtooth spikes with 10, 20, 40 μm spacings (S-10, S-20, S-40), respectively.

High-magnification imaging was employed to analyze the contact line dynamics during the self-propulsion of a single barrel droplet (excluding coalescence-induced propulsion) (Movies S4–S5). For the contact line snapping at the front (θ_*Front*_ to θ_*Adv*._), all sawtooth spikes exhibited a θ_*Front*_ of 35 ± 3°. For the S-20 and S-40 spikes, the corresponding θ_*Adv*._ values were approximately 25° and 15°, respectively. Due to the small teeth spacing and irregular surface structure, it was challenging to accurately measure the contact angle (θ_*Adv*._) for the S-10 spike. Consequently, it was estimated to be 28° based on the geometric principles of a right triangle, as illustrated in Figure S13. The tensioning of the rear contact line (θ_*Rear*_ to θ_*Rec*._) for all spikes exhibited a change in angle from 18° to approximately 0°, corresponding to the receding contact angle of water on a 3D printed flat surface (Figure S14c). Overall, the observed changes in contact line angle resulted in a net hysteresis force per unit width per tooth that was highest for the S-40 spike, followed by S-20 and S-10 (Figure 5c).

A barrel droplet undergoing self-propulsion initially exhibits a capillary force that surpasses the hysteresis force exerted by the teeth. As it propels, the droplet encounters enough teeth for the hysteresis force to reach a magnitude comparable to the capillary force. The hysteresis force per tooth increases proportionally with the spike’s axial width. Incomplete wetting ahead of the droplet may necessitate a larger surface area and capillary force to initiate the self-propulsion, leading to the droplet sweeping a greater number of teeth. Therefore, the capillary force exerted on the barrel droplet immediately before movement (*F*_*nc*_) is directly proportional to the product of spike width (*r*) and number of teeth swept during its self-propulsion (*N*). We recorded 10 self-propulsion events for each sawtooth spike, and the results show the predicted values of *r* · *N* in eq 9 agree well with the captured values from footage for the S-20 and S-240 spikes. However, for the S-10 spike, the predicted values exhibited slight deviations compared to the measured values, likely due to the pronounced randomness and irregularity of its sawtooth microstructures (Figure 5d).

$$\tau_{Front/Rear}=\gamma (\cos\theta_{Adv./Rec.}-\cos\theta_{Front/Rear})$$ 7

$$F_{h}=2{\pi r}^{*}\left(\tau_{Front}-\tau_{Rear}\right)$$ 8

$$r\cdot N=F_{nc}/2\pi \left(\tau_{Front}-\tau_{Rear}\right)$$ 9

An array of spikes was fabricated to study the overall fog harvesting performance of the 3D printed sawtooth spikes. The schematics in Figure 5e show that there is an extended flat surface under the array of sawtooth spikes to facilitate water removal (Figure S15b). The results show that without the presence of an extended surface beneath the spike array, harvested water adheres to the spikes, preventing water harvesting (Figure S15c). Moreover, during the initial phase of droplet movement, the droplets grow larger as they reach the base of the spikes. Following the initial movement of the barrel droplets, the spikes become fully wetted.^29^ At this stage, the droplets reaching the base tend to be smaller in size (Figure S15a). After reaching the spike base, water fills the extended surface until gravity forces a macro-droplet of water (∼50 mg) to detach. The residual water film adhered to the surface acts as a lubricating layer for subsequent droplets from the spike array until another macro-droplet detachment (Figure S15d and Figure 5f). Additionally, altering the spacing between teeth significantly impacts the fog harvesting rate. The S-10 spike exhibits a fog harvesting rate (∼333 mg/cm^2^.hr) nearly double that of the S-20 and triple that of the S-40. This confirms that small and dense sawtooth surface structures can enhance fog harvesting performance, as shown in Figure 5f.

For large water droplets, we have studied their gravity-driven motions to reveal how sawtooth or imbricated surface structure alone affects the movement. To isolate the effect of surface structure from spike curvature, flat surfaces with sawtooth and imbricated structures were fabricated via 3D printing and vertically mounted for gravity-driven droplet sliding experiments. Our results in Figure 6 show that droplets on surfaces with sawtooth and imbricated structures began to slide with very different apparent advancing contact angles though the advancing contact angle of flat surface without structure is around 52 ± 5° (Figure S14b). Under the Wenzel wetting regime, the behavior of the contact line is crucial as it progresses over surface structure. On the dry surface with sawtooth structure, the leading edge of the droplet crosses the tip of a tooth and subsequently moves over a surface inclined at 10° relative to the vertical direction of gravitational force, as shown in Figure 6a. The droplet meniscus exhibits an apparent advancing contact angle (θ_*A*, *flat*_) of approximately 62°, in contrast to the intrinsic contact angle of 52° (Figure 6b). Conversely, on the dry surface with imbricated structure as shown in Figure 6d, the front contact line advances over a surface oriented horizontally after surpassing the tooth tip, exhibiting a significantly higher apparent contact angle of approximately 146° (Figure 6e). Even when the surfaces were prewetted, the sawtooth structure exhibited a lower advancing contact angle of 45° (Figure 6c), whereas the imbricated structure maintained a much higher contact angle of 80° (Figure 6f). Moreover, our measurements demonstrate that on the sawtooth surface, droplet sliding commenced at a critical mass of roughly 20 mg, corresponding to a contact angle hysteresis force of approximately 37 dyn/cm, while this force decreased to 18 dyn/cm under wet conditions. In comparison, on the imbricated surface under dry conditions, the droplet required a mass of 31 mg to initiate sliding, correlating to a notably higher hysteresis force per unit length (*F*_*h*, *flat*_) as in eq 10 of 131.65 dyn/cm. Under wet conditions, this hysteresis force decreased to 57 dyn/cm, and the critical sliding mass was substantially reduced to 8 mg (Figure 6h). Overall, these findings reveal the unique role of the sawtooth surface structure in promoting droplet mobility. Combining the structure with the conical spike curvature allows water to be retained between the gaps of the teeth more effectively, which helps maintain a low contact angle hysteresis. This, in turn, enhances droplet propulsion along the sawtooth conical spike.

$$F_{h,flat}=\gamma (\cos\theta_{R,flat}-\cos\theta_{A,flat}),CAH=\theta_{A,flat}-\theta_{R,flat}$$ 10

where θ_*R*, *flat*_, θ_*A*, *flat*_ are the apparent receding and advancing contact angles of flat surface with sawtooth or imbricated structure.

**Figure 6:**
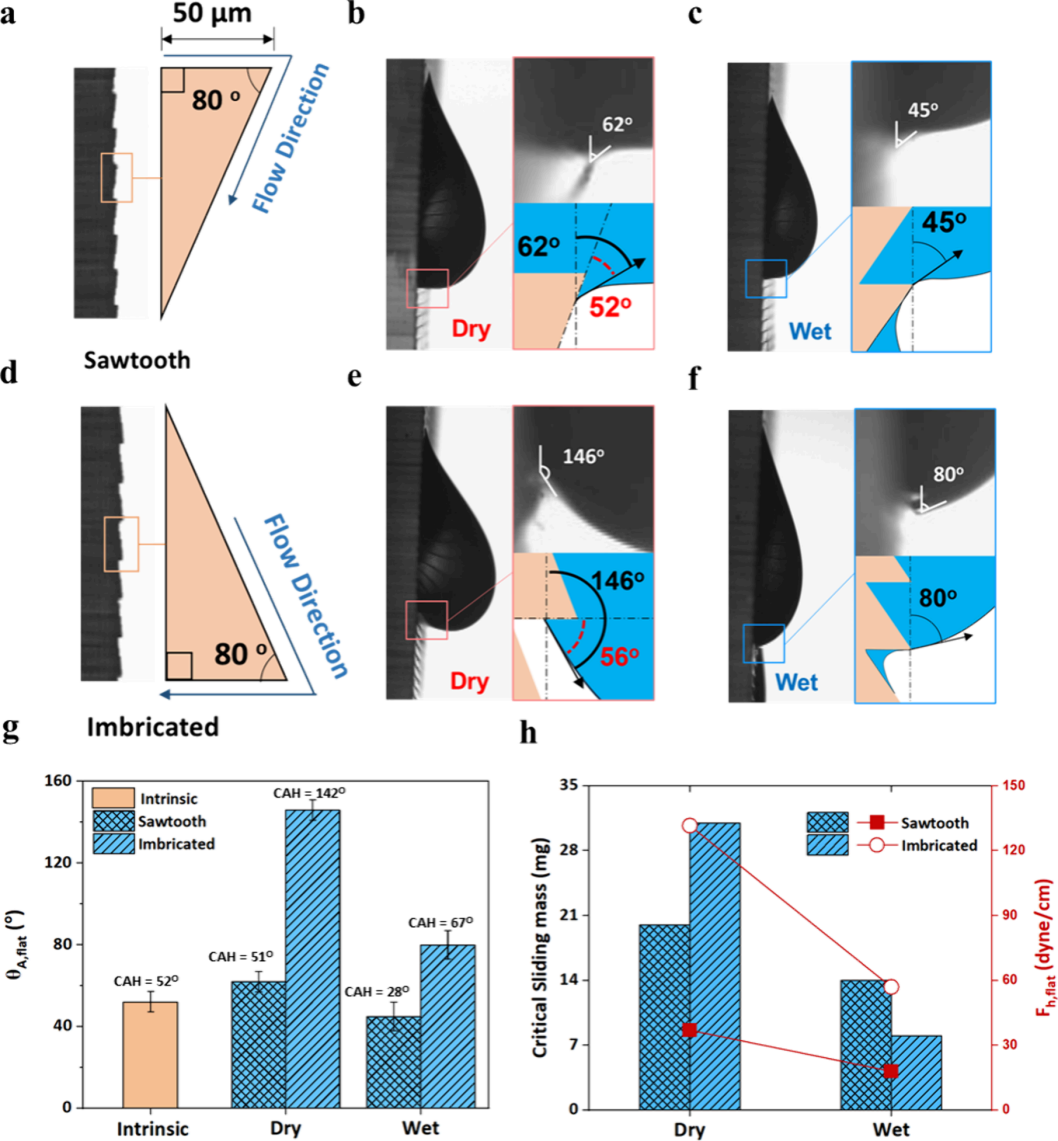
Droplet dynamics on structured flat surfaces. Optical images and schematic illustrations of the tooth geometries for both (a) sawtooth and (d) imbricated structures, highlighting the flow direction as the droplet’s contact line advances past the tooth tip. Advancing meniscus on the surface with sawtooth structure under (b) dry and (c) wet conditions. Advancing meniscus on the surface with imbricated structure under (e) dry and (f) wet conditions. The angles indicated by the black solid line show the apparent advancing contact angles (θ_*A*, *flat*_), while the red dashed line represents the intrinsic contact angle relative to the local surface. (g) Advancing contact angle and contact angle hysteresis of flat surfaces without structure and with sawtooth and imbricated structures. (h) Critical sliding mass in mg and hysteresis force (*F*_*h*, *flat*_) in dyn/cm for flat surfaces with sawtooth and imbricated structures under dry and wet conditions.

## Conclusion

To sum up, this study has revealed the mechanism of self-propelling droplets along shape-gradient spikes for bioinspired fog harvesting. Conical spikes with microscale sawtooth and imbricated surface structures under variable teeth spacing were fabricated with high-resolution 3D printing. The sawtooth spikes outperformed those with smooth and imbricated structured surfaces by producing small and mobile barrel droplets. Our findings demonstrate that the orientation of surface structures relative to the fog stream, along with the difference in teeth-gap area, significantly influences the directional droplet transport toward spike base. Sawtooth structures enable the water to fill the gaps among the teeth of wettable spikes, while the resulting barrel droplets get pinned by the dry top edges of sawtooth. Given the high energy state of the barrel droplet, we observed a decrease in surface energy as the droplet grew and moved along the conical spike. The shape of mobilized barrel droplets flattens while occupying a larger conical solid area. Accordingly, its surface energy budget and capillary pressure difference diminish, causing a decline in movement and eventual stagnation when the gravity force dominates. Our analysis of self-propulsion events has revealed that smaller sawtooth gaps on the spike surface enhance directional droplet transport and improve fog harvesting efficiency. A uniformly structured array of 10 μm-spacing spikes yielded a fog collection rate (∼333 mg/cm^2^.hr) that was almost twice that of the array with 20 μm-spacing and three times that of the array with 40 μm-spacing. Our in-depth analysis of large droplet transport on flat surfaces with sawtooth and imbricated structures further demonstrates that the imbricated tooth exerted 3.5 times the hysteresis force on moving droplets compared to the sawtooth under dry conditions, and almost twice the hysteresis force under wet conditions. In summary, these findings highlight the advantage of integrating sawtooth surface structures on conical spikes to achieve high-performance fog harvesting and passive directional droplet transport.

## Methods

### Spikes Fabrication

High-resolution 3D printer (Boston Micro Fabrication, S230) was used to print sawtooth and imbricated spike structures. The printer’s top-down printing process, combined with the resin dye’s effect on the UV spot size, resulted in a cured layer with a larger cross-section at the top and a gradually decreasing cross-section toward the base. This created a series of evenly spaced sawtooth protrusions on the spike’s surface. To fabricate spikes with a reversed sawtooth orientation (imbricated), we inverted the printing orientation and exploited a weight-induced bending phenomenon to create the desired imbricated pattern. For the smooth spikes, we coated glass spikes in a diluted 3D printing resin solution with iso-propanol. After dipping the spikes, we placed them in a UV oven to cure the resin. Finally, we cleaned the coated spikes with ethanol to remove any excess uncured resin, resulting in a smooth, resin-coated surface.

### Characterization

High-resolution optical imaging system (Phantom, model: MIRO M310) was used to record the time-lapse images with a temporal resolution of 24 frames per second. Equipped with a 5x lens, we captured the overall journey of the droplets on the conical surfaces. To capture the droplet dynamics, 10x and 50x lenses were used. Scanning electron microscopy (Quanta 250, SEM) was utilized to examine the microstructure of the spikes. Contact angle goniometer (FAMAS) to measure both static and dynamic contact angles.

## Supplementary Material













## Data Availability

The paper and its Supporting Information provide all the data required to assess our findings. Additional data related to this research is available upon request.

## Notes

Optical images of the wetting of teeth-gaps during fog and vapor exposure on sawtooth and imbricated spikes; time-lapsed footage of initial stages of fog harvesting on the sawtooth spike; coordinates of the barrel droplets after self-propulsion as a function of droplet spherical radius; high-resolution 3D printing of the Sawtooth spikes; high-resolution 3D printing of the Imbricated spikes; droplet dynamics on imbricated spikes versus sawtooth spikes; fog harvesting on superhydrophilic glass spike; preparation and droplet formation on the smooth spike; experimental setup and fog stream characteristics; comparison between measured and calculated principal radius of curvature at the rear side of the droplets (*R*_*t*_^1^); time-lapsed images of a microdroplet dispensed on (a) wet and (b) dry sawtooth spikes; optical images of fog harvesting on sawtooth conical spikes with different cone apex angels; schematic of the geometric principle of a right triangle utilized to predict the θ_Adv._ of the droplet on the S-10 spike; wettability characteristics of a 3D printed flat surface without structure; sawtooth spike array for fog harvesting (PDF)
Droplet dynamics on the sawtooth spike during fog harvesting (MP4)
Droplet dynamics on the imbricated spike during fog harvesting (MP4)
Droplet stagnation on the smooth spike during fog harvesting (MP4)
Contact line snapping at the front side (MP4)
Contact line tensioning at the rear side (MP4)
