# Supplementary material for: Self-Propelling Water Droplets on Conical Spikes with Sawtooth Surface Structure
Source: ACS Appl Mater Interfaces. 2025 May 16;17(21):31604–13. doi: 10.1021/acsami.5c03846 (PMC12123620; doi:10.1021/acsami.5c03846)
Supplement: Supplementary file 1 [file am5c03846_si_001.pdf]

Supporting Information

Self-Propelling Water Droplets on Conical Spikes with Sawtooth Surface Structure

Abubaker S. Omer, Aikifa Raza and TieJun Zhang*

Department of Mechanical and Nuclear Engineering, Khalifa University of Science and Technology, P.O. Box 127788, Abu Dhabi, United Arab Emirates

*Corresponding author. Email: tiejun.zhang@ku.ac.ae

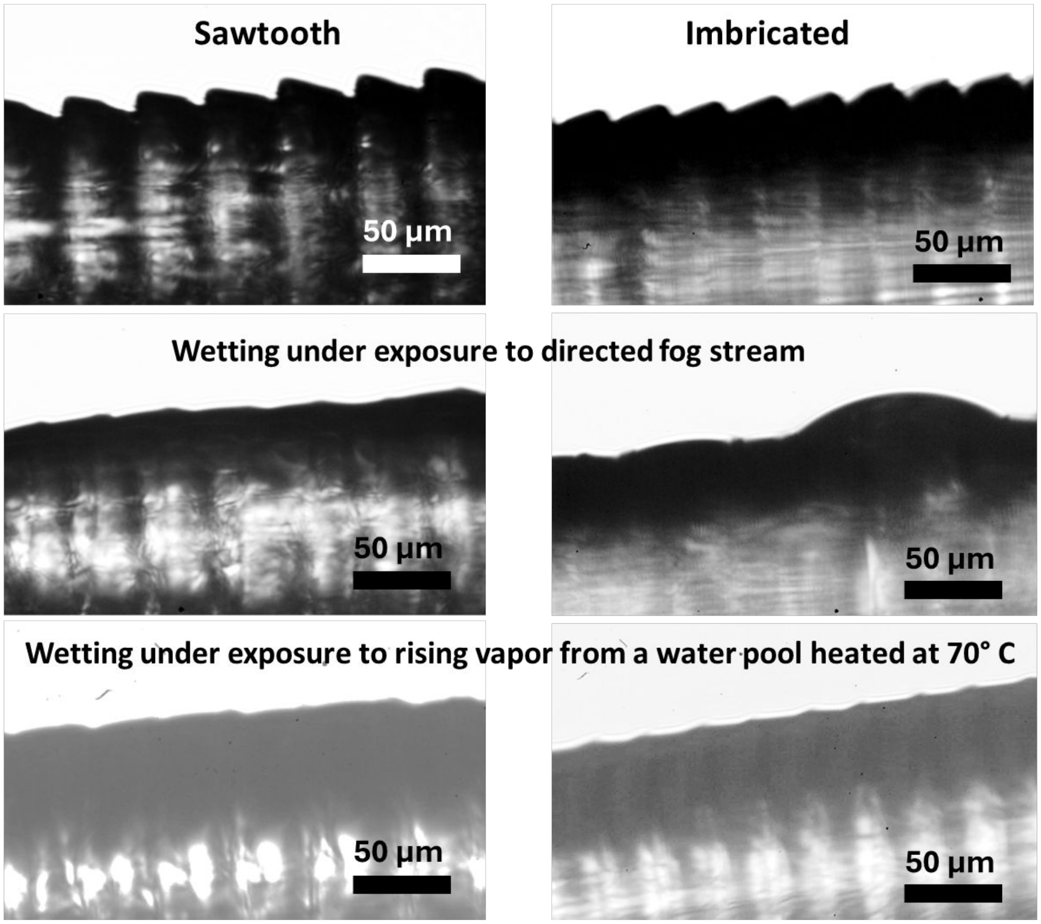

Figure S1. Optical images of the wetting of teeth-gaps during fog and vapor exposure on sawtooth and imbricated spikes.

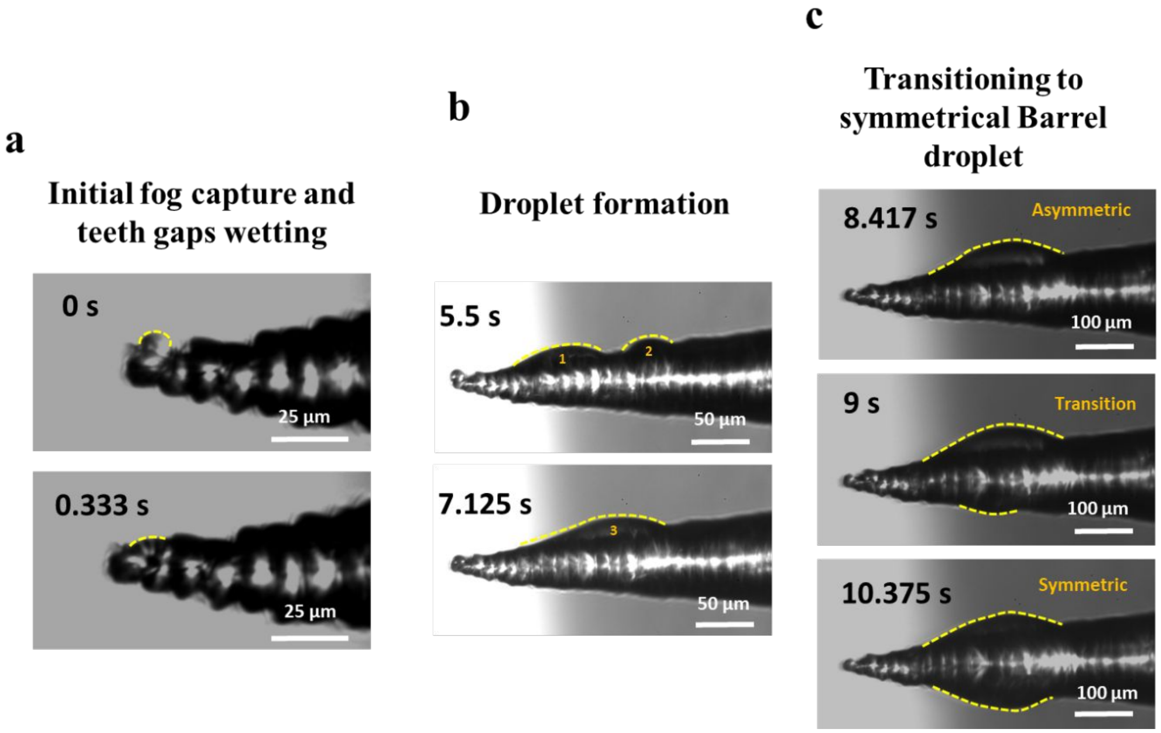

Figure S2. Time-lapsed footage of initial stages of fog harvesting on the sawtooth spike. (a) fog capture and wetting of the teeth gaps. (b) Asymmetrical barrel droplet formation and coalescence. (c) Shape transition from asymmetrical to symmetrical barrel droplet.

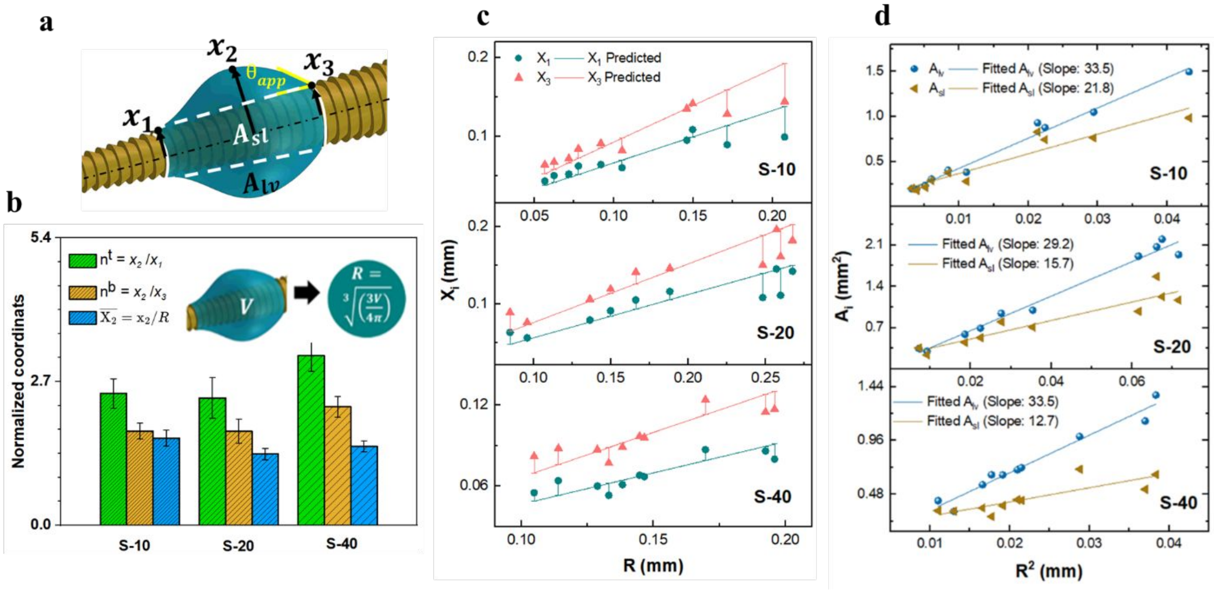

Figure S3. Coordinates of the barrel droplets after self-propulsion as a function of droplet spherical radius. (a) Schematic of coordinates and apparent contact angle. (b) Normalized coordinates $\boldsymbol{n^t = x_2/x_1}$, $\boldsymbol{n^b = x_2/x_3}$ and $\boldsymbol{\bar{X}_2 = x_2/R}$ of the barrel droplets after self-propulsion. (c) Measured and predicted coordinates (x_1 and x_3) and (d) surface areas ($\boldsymbol{A_{lv}}$ and $\boldsymbol{A_{sl}}$).

We employed the analytical solution developed by Michielsen et al.[1] *to* quantify the three-dimensional liquid-vapor surface area (A_{lv}) and volume (V) of symmetrical barrel droplets enveloping the sawtooth spikes. The independent variables of the model are the droplet coordinates (x_1, x_2 *and* x_3) and the contact angle (θ_{app}).

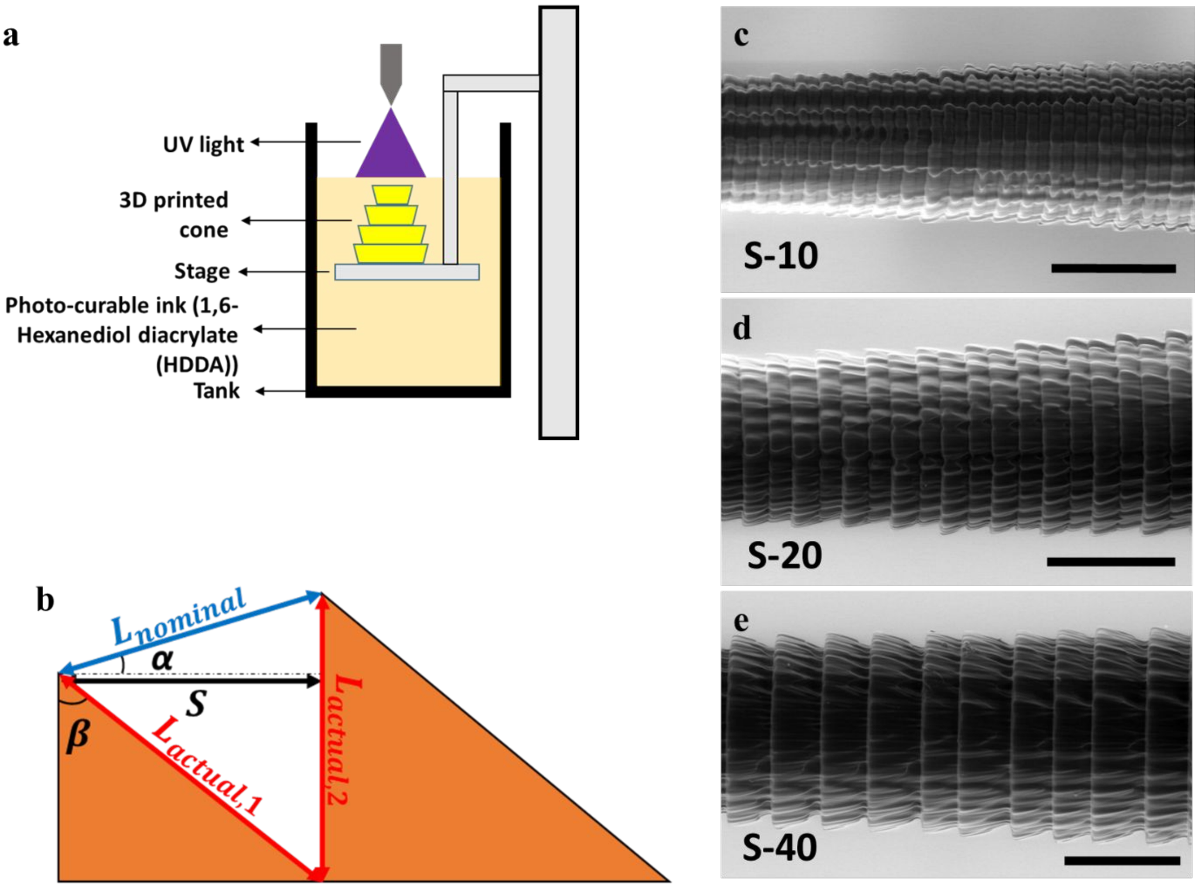

Figure S4. High-resolution 3D printing of the Sawtooth spikes. (a) Schematic of the stereolithographic 3D printing setup, (b) schematic of the saw-tooth microstructure, and SEM imaging of the (c) S-10, (d) S-20, and (e) S-40 sawtooth spikes with scale bars at 100 μm.

By utilizing high-resolution (10 μm) **s**tereolithographic 3D printing, the sawtooth spikes were intrinsically generated. The 3D printer operates on a top-down printing scheme, where the stage descends, and photo-crosslinking initiates from the top of the printing layer, progressing downward. Owing to the presence of the resin dye, the UV spot size diminishes throughout the thickness of the printing layer, resulting in a cured layer with a larger cross-section at the top that gradually reduces in size along the layer thickness. Ultimately, the 3D printing operation of a spike will result in a surface featuring sawtooth-like microstructures that are equally spaced. An analysis of the surface area ratio between saw-tooth spikes ($Saw-teeth\ A_{sl}$) and smooth spikes ($Smooth\ A_{sl}$), revealed that it is independent of teeth spacing. Using ImageJ software, the (β) angles of all spikes were found to be equal 79 $\pm 5^o$ and the ratio of the surface areas consistently approached 1.25.

$$\frac{Saw-teeth\ A_{sl}}{Smooth\ A_{sl}} = \frac{L_{actual}}{L_{nominal}} = \cos(\alpha) * \left[\frac{1+\sin(90-\beta)}{\cos(90-\beta)} + \tan(\alpha)\right] \approx 1.25 \quad \text{(S1)}$$

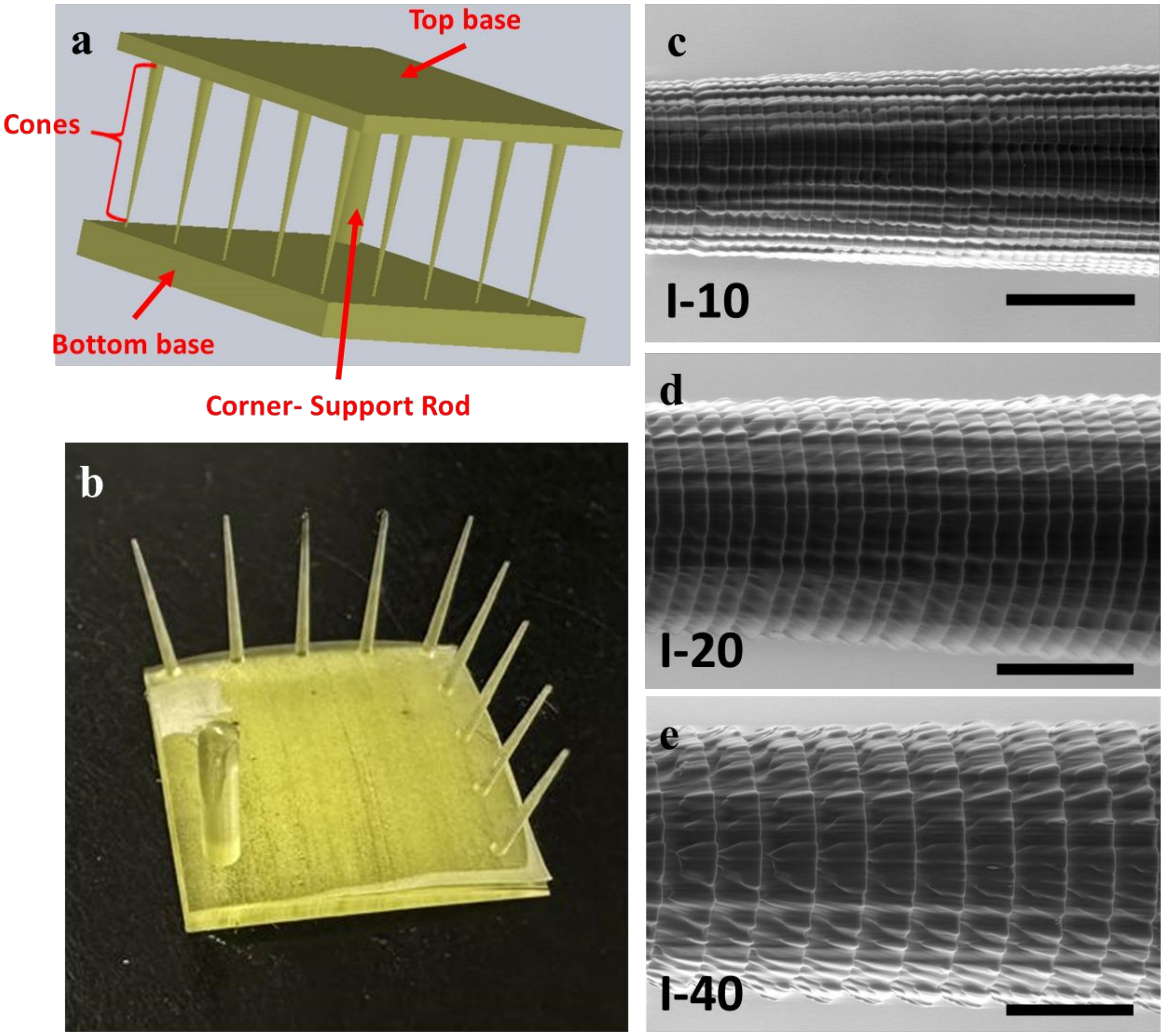

Figure S5. High-resolution 3D printing of the Imbricated spikes. (a – b) The CAD model and 3D printed collection of imbricated spikes. (c – e) SEM images of the 3D printed imbricated spikes with three teeth spacings (10, 20, and 40 µm) with scale bars at 100µm.

To fabricate reversed sawtooth orientation (imbricated) micro-structures, spikes were printed upside-down, exploiting a weight-induced bending phenomenon during fabrication. As the top-down printing operation reaches the top base layers of the model, the weight of the base causes the entire structure, supported by the spike tips and a corner-support rod, to bend slightly. This bending resulted in the spike tips breaking cleanly from the bottom base and tilting outwards (Figure S5b). The corner rod is then broken manually, allowing the imbricated spikes (I-10, I-20, and I-40) to be used for studying droplet dynamics during fog harvesting.

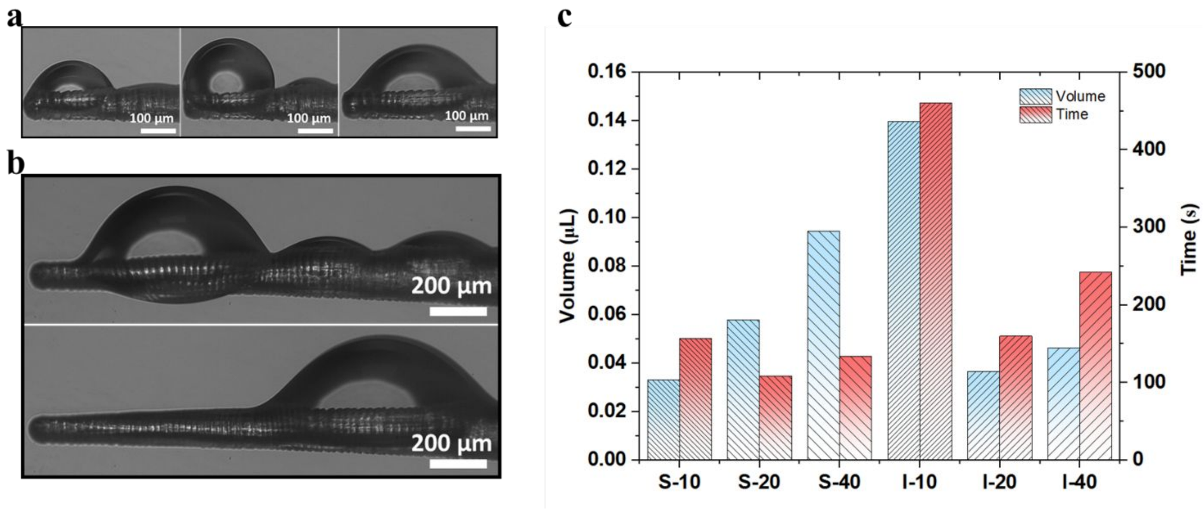

Figure S6. Droplet dynamics on imbricated spikes versus sawtooth spikes. (a) Initial droplet growth on an imbricated spike during fog harvesting. (b) Coalescence-induced self-propulsion of a droplet on an imbricated spike. (c) Volume and time required for a barrel droplet to reach a spike radius of 155 ± 3 µm at the droplet's apex on both sawtooth and imbricated spikes.

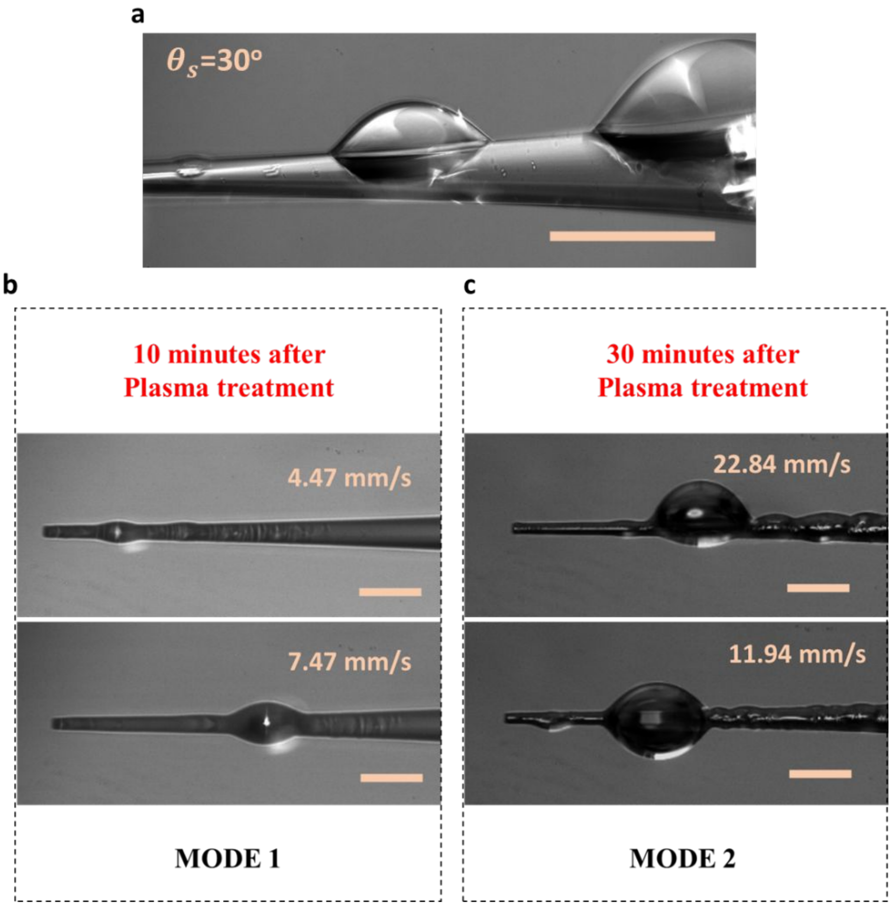

Figure S7. Fog harvesting on superhydrophilic glass spike. (a) intrinsic water contact angle on the untreated glass spike. Modes of droplet transport on the plasma treated glass spike as the contact angle of water increases from (b) superhydrophilicity to the (c) intrinsic water contact angle of the glass spike. Scale bars at 1 mm.

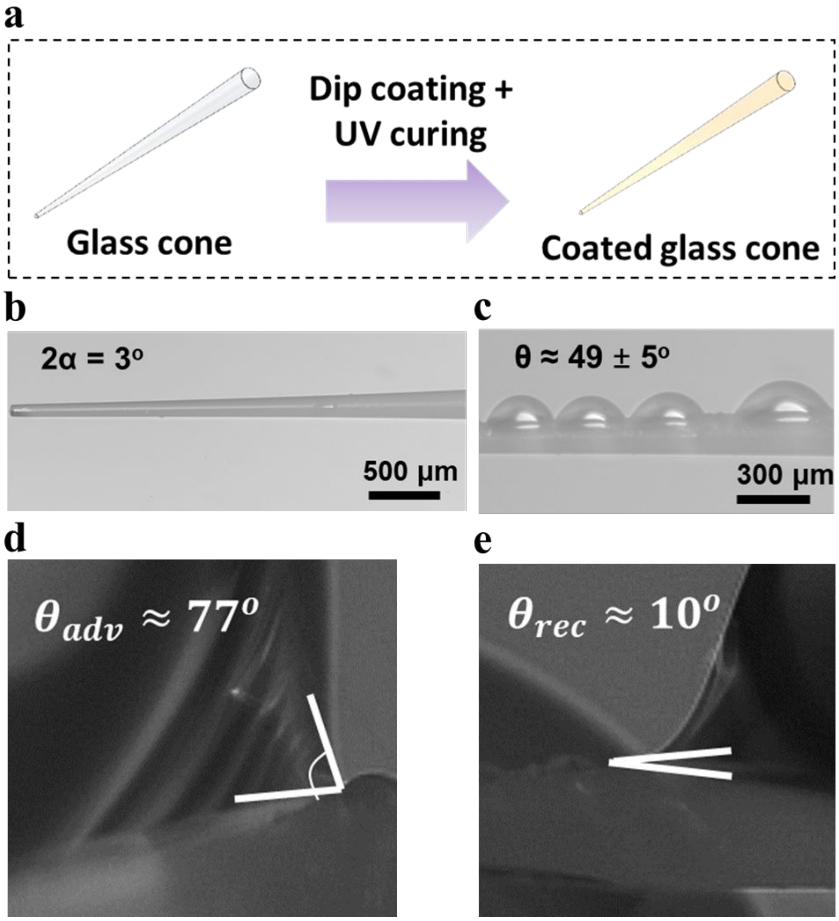

Figure S8. Preparation and droplet formation on the smooth spike. (a) Schematic of the smooth spike preparation method. (b) The smooth shows an apex angle of 3°. (c) Contact angle of water at early stages of fog harvesting. (d) Advancing and (e) receding contact angles.

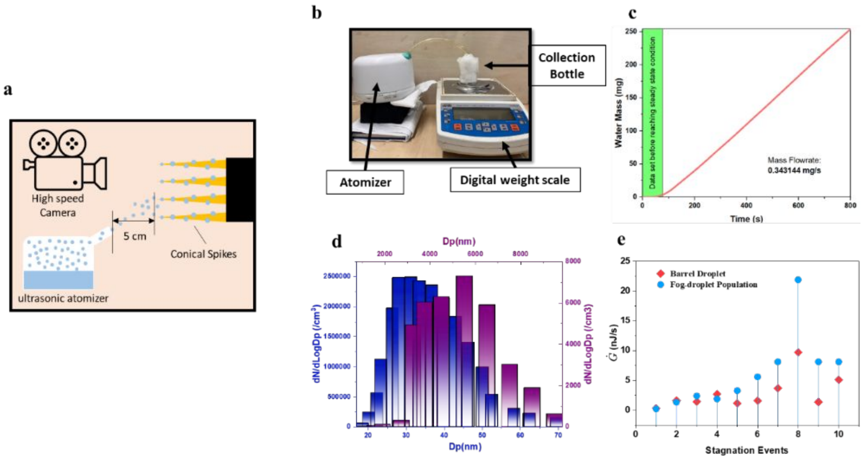

Figure S9. Experimental setup and Fog stream characteristics. (a) Fig harvesting setup**.** (b – c) Experimental set-up to measure the atomizer mass flowrate (0.34 mg/s). (d) Size distributions of pure water mist generated at 2.4 MHz frequency as reported by Kudo et al.[2]. (e) Energy growth rate of barrel droplets approaching the energy addition rate of the condensing fog stream population.

The fog harvesting setup comprises an enclosed chamber made from plastic sheets, housing the high-speed camera, fog generator, and the test spikes. This design ensures containment of the fog stream while allowing for controlled exhaust. The environmental conditions within the enclosed chamber were at a constant range of 50 – 60% relative humidity and 22 – 24 °C. The fog generator was positioned at a fixed distance of 5 cm from the conical spikes to ensure consistent exposure. Using the reported size distribution data for pure water mist generated at 2.4 MHz frequency by Kudo et al. (*37*), we calculated the number density per cubic millimeter by converting the logarithmic distribution to number/ mm^3 distribution (Eq. S.2). By considering a fog cluster as the fog population within a 1 mm^3 volume of air, we determined the rate of fog clusters coalescing with the barrel droplet during a stagnation period ($\dot{NC}$) (Eq. S.3) based on the barrel droplet's mass change. Subsequently, we calculated the rate of energy addition to the barrel droplet due to these coalescing fog clusters (Eq. S.4).

$$\frac{dN}{dLog d_p} = 2.303 * \frac{F_{m0}}{\sqrt{2\pi}\ln\sigma_g} \times \exp\left[-\frac{(\ln d_p - \ln d_{g0})^2}{2(\ln\sigma_g)^2}\right] \quad (S2)$$

$$\dot{NC} = \left(\frac{\Delta m}{\Delta t}\right)_{Barrel} \Big/ \left[\rho * \sum_{rmin}^{rmax} \frac{N(r_i)\,4}{3}\pi\,[r_i^{6}] * V_{Barrel}\right] \quad (S3)$$

$$\dot{G}_{Fog-droplet\ population} = NC * \gamma * \sum_{rmin}^{rmax} N(r_i)\,4\pi[r_i^{2}] * V_{Barrel} \quad (S4)$$

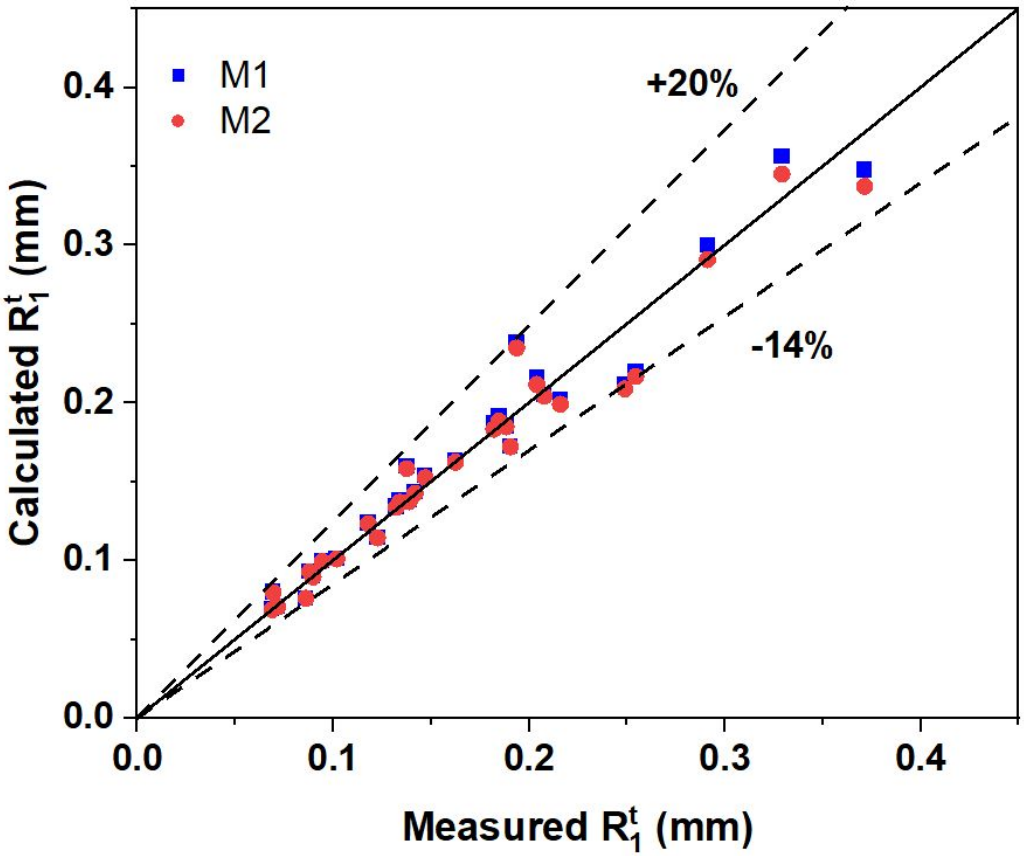

Figure S10. Comparison between measured and calculated principal radius of curvature at the rear side of the droplets ($R^t{}_1$).

M1[1] : $\Delta P_c^t = \frac{2\gamma\left[x_1 cos(\alpha+\theta)-x_2\right]}{x_1^2-x_2^2} = \gamma\left(\frac{1}{x_1} - \frac{1}{\boldsymbol{R_1}^{\boldsymbol{t}}}\right)$ (S5)

M2[3] : $\Delta P_c^t = \frac{2\gamma}{x_2+x_1} = \gamma\left(\frac{1}{x_1} - \frac{1}{\boldsymbol{R_1}^{\boldsymbol{t}}}\right)$ (S6)

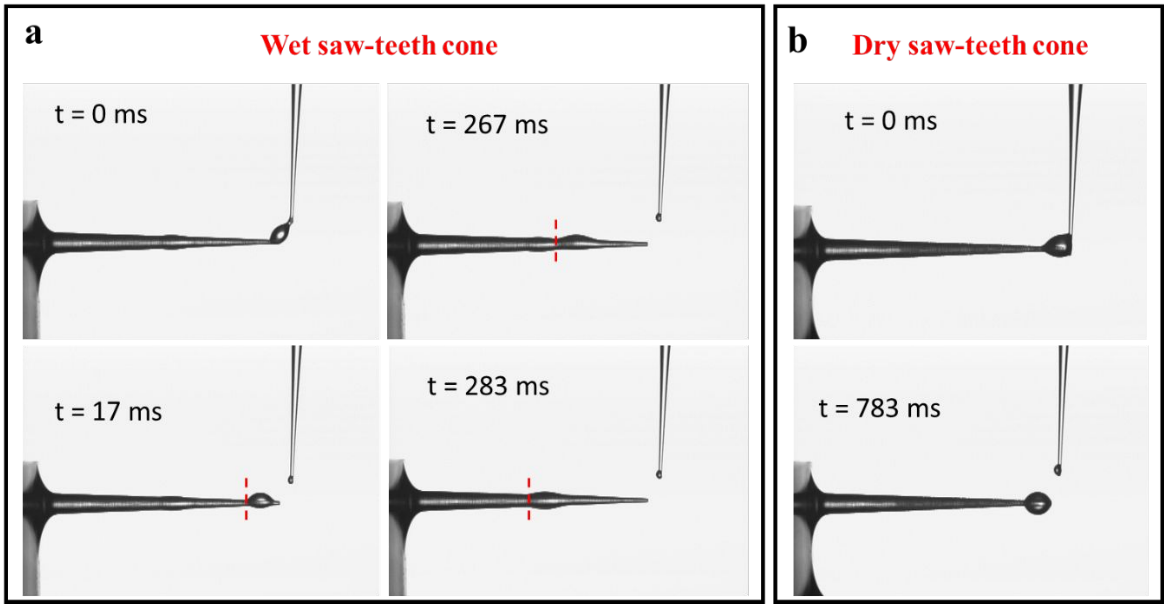

Figure S11. Time-lapsed images of a micro-droplet dispensed on (a) wet and (b) dry sawtooth spikes.

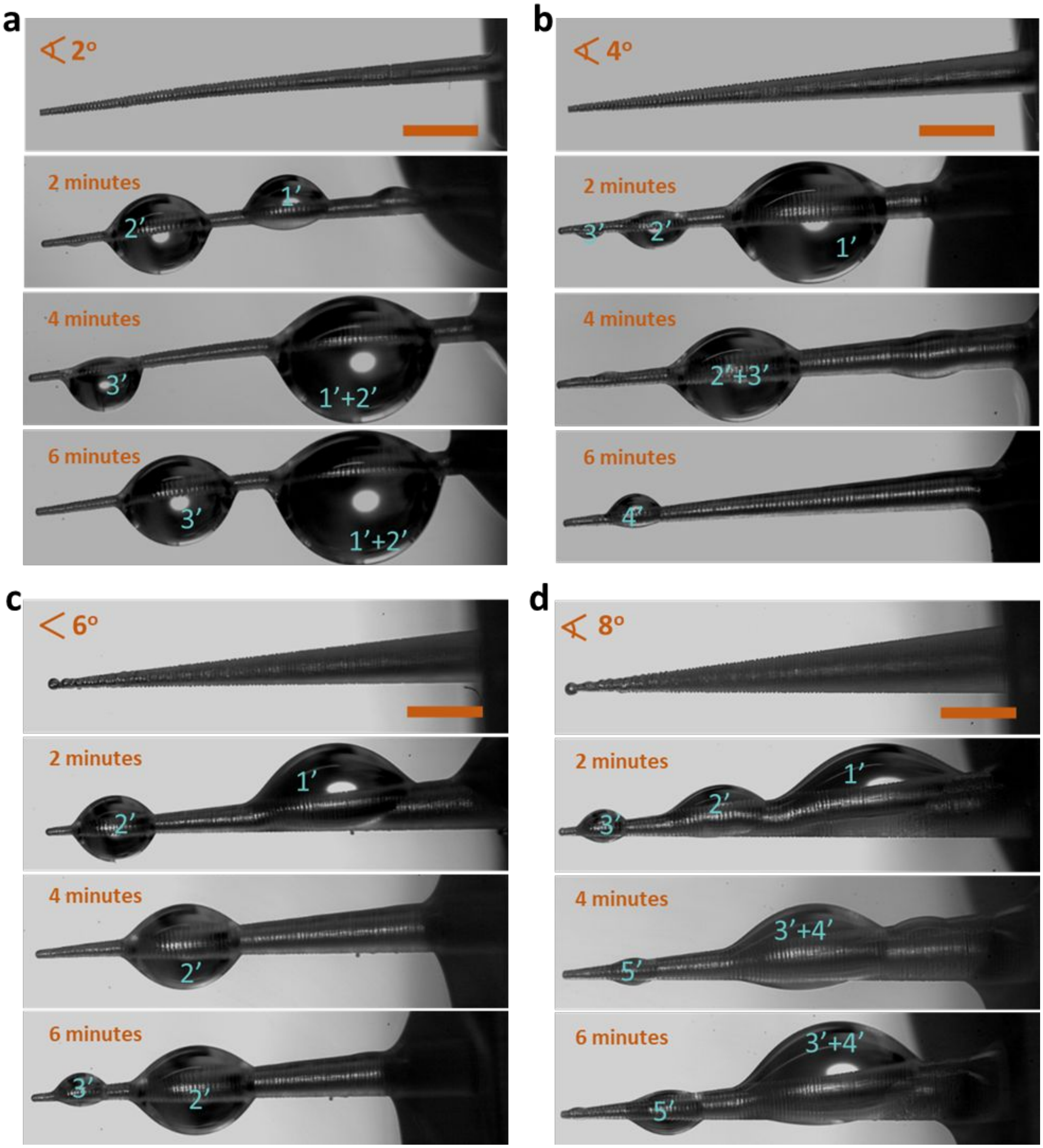

Figure S12. Optical images of fog harvesting on sawtooth conical spikes with cone apex angels (2α) of (a) 2, (b) 4, (c) 6 and (d) 8° with scale bars at 1mm.

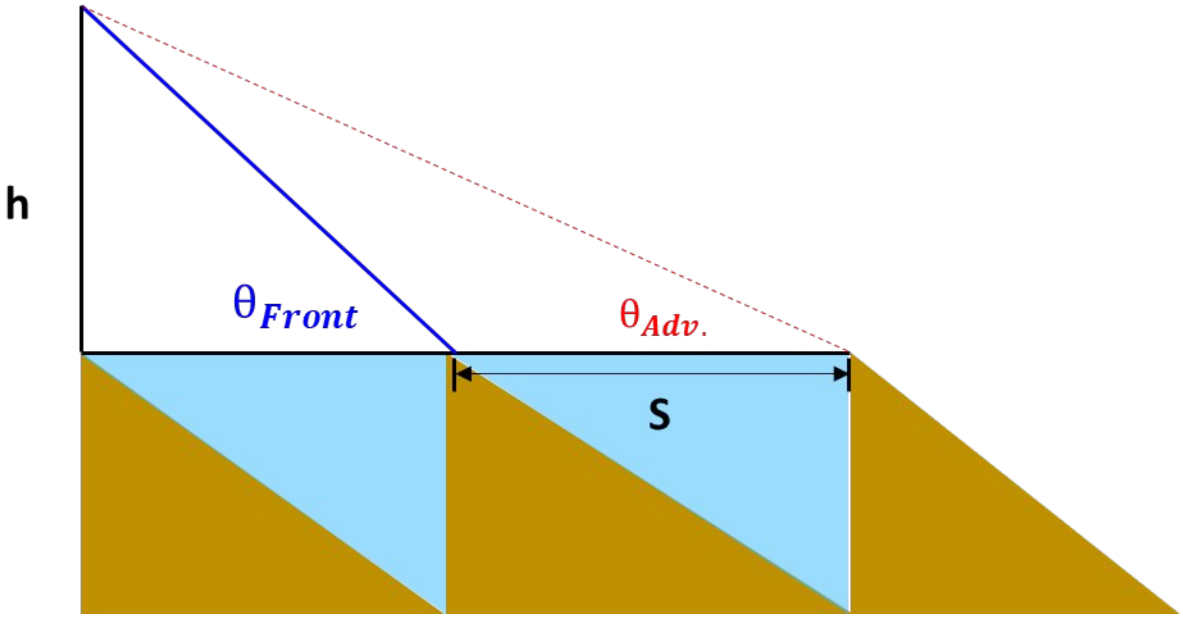

Figure S13. Schematic of the geometric principle of a right triangle utilized to predict the $\boldsymbol{\theta_{Adv.}}$ of the droplet on the S-10 spike.

Based on the theoretical value of h for the S-20 spike, the contact angle ($\boldsymbol{\theta_{Adv.}}$) was approximated to 28°.

$$\boldsymbol{h\ (S-20) = S * \left[\frac{tan(\theta_{Front}) * tan(\theta_{Adv.})}{tan(\theta_{Front}) - tan(\theta_{Adv.})}\right] = 26\ \mu m} \quad \text{(S7)}$$

$$\boldsymbol{\theta_{Adv.}\ (S-10) = tan^{-1}\left[\frac{h * tan(\theta_{Front})}{h + (S * tan(\theta_{Front}))}\right] \approx 28^{o}} \quad \text{(S8)}$$

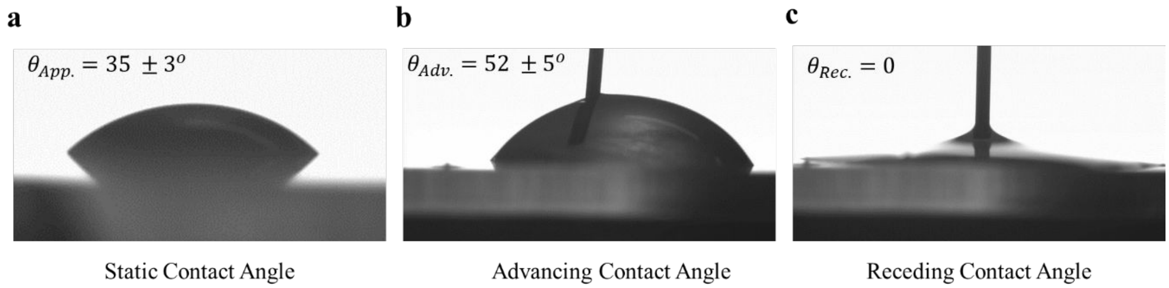

Figure S14. Wettability characteristics of a 3D-printed flat surface without structure. (a) Static, (b) Advancing and (c) Receding contact angles.

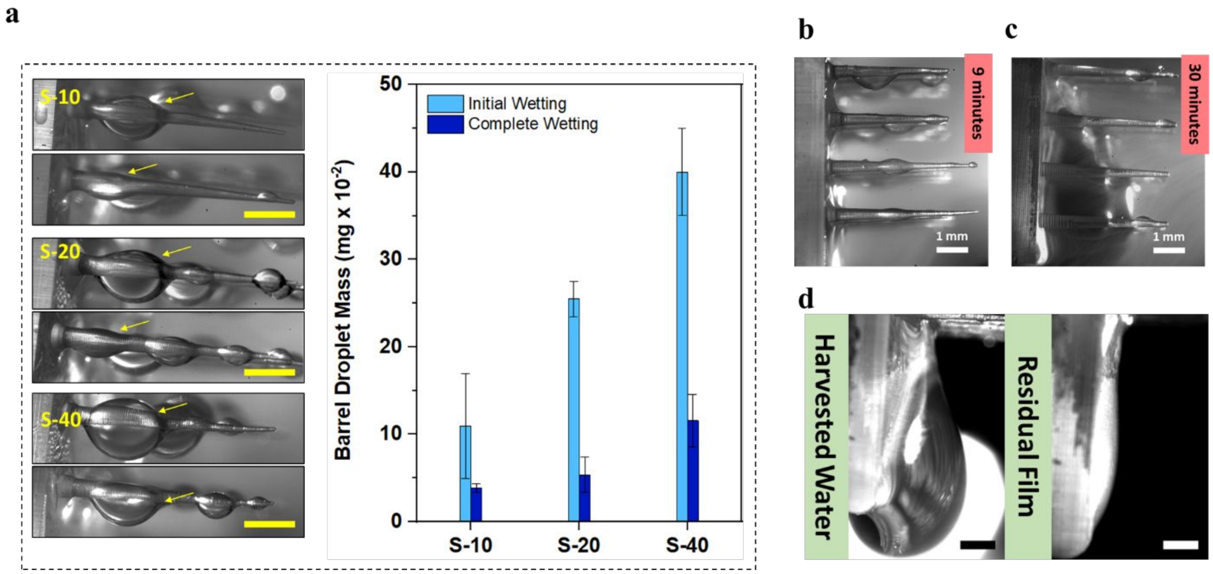

Fig. S15. Sawtooth spike array for fog harvesting. (a) Droplet mass at the spike base during initial and complete wetting. (b) Array of spikes with an extended surface that enables water harvesting in contrast to (c) an array without an extended surface, demonstrating water entrapment and prevention of harvesting. (d) Harvested water and residual film post harvesting. All scale bars at 1mm.

Movie S1. Droplet dynamics on the sawtooth spike during fog harvesting.

Movie S2. Droplet dynamics on the Imbricated spike during fog harvesting.

Movie S3. Droplet stagnation on the smooth spike during fog harvesting.

Movie S4. Contact line snapping at the front side.

Movie S5. Contact line tensioning at the rear side.

Supplementary References

(1) Michielsen, S.; Zhang, J.; Du, J.; Lee, H. J. Gibbs Free Energy of Liquid Drops on Conical Fibers. *Langmuir* **2011**, *27* (19), 11867–11872.

(2) Kudo, T.; Sekiguchi, K.; Sankoda, K.; Namiki, N.; Nii, S. Effect of Ultrasonic Frequency on Size Distributions of Nanosized Mist Generated by Ultrasonic Atomization. *Ultrason. Sonochem.* **2017**, *37*, 16–22.

(3) de Gennes, P.-G.; Brochard-Wyart, F.; Quéré, D. Capillarity: Deformable Interfaces. In *Capillarity and Wetting Phenomena*; Springer New York, 2004; pp 1–31.
